# Radiomic signature of the FOWARC trial predicts pathological response to neoadjuvant treatment in rectal cancer

**DOI:** 10.1186/s12967-021-02919-x

**Published:** 2021-06-10

**Authors:** Zhuokai Zhuang, Zongchao Liu, Juan Li, Xiaolin Wang, Peiyi Xie, Fei Xiong, Jiancong Hu, Xiaochun Meng, Meijin Huang, Yanhong Deng, Ping Lan, Huichuan Yu, Yanxin Luo

**Affiliations:** 1 Department of Colorectal Surgery, Sixth Affiliated Hospital, Sun Yat-sen University, 26 Yuancun Erheng Road, Guangzhou, 510655 Guangdong China; 2 Guangdong Institute of Gastroenterology, Guangdong Provincial Key Laboratory of Colorectal and Pelvic Floor Disease, Sixth Affiliated Hospital, Sun Yat-sen University, 26 Yuancun Erheng Road, Guangzhou, 510655 Guangdong China; 3 Department of Biostatistics, Columbia University, New York, NY 10032 USA; 4 Department of Radiology, Sixth Affiliated Hospital of Sun Yat-sen University, Guangzhou, 510655 Guangdong China; 5 Department of Medical Oncology, Sixth Affiliated Hospital, Sun Yat-sen University, Guangzhou, 510655 Guangdong China

**Keywords:** Radiomics, Computed tomography, Neoadjuvant treatment, Rectal cancer

## Abstract

**Background:**

We aimed to develop a radiomic model based on pre-treatment computed tomography (CT) to predict the pathological complete response (pCR) in patients with rectal cancer after neoadjuvant treatment and tried to integrate our model with magnetic resonance imaging (MRI)-based radiomic signature.

**Methods:**

This was a secondary analysis of the FOWARC randomized controlled trial. Radiomic features were extracted from pre-treatment portal venous-phase contrast-enhanced CT images of 177 patients with rectal cancer. Patients were randomly allocated to the primary and validation cohort. The least absolute shrinkage and selection operator regression was applied to select predictive features to build a radiomic signature for pCR prediction (rad-score). This CT-based rad-score was integrated with clinicopathological variables using gradient boosting machine (GBM) or MRI-based rad-score to construct comprehensive models for pCR prediction. The performance of CT-based model was evaluated and compared by receiver operator characteristic (ROC) curve analysis. The LR (likelihood ratio) test and AIC (Akaike information criterion) were applied to compare CT-based rad-score, MRI-based rad-score and the combined rad-score.

**Results:**

We developed a CT-based rad-score for pCR prediction and a gradient boosting machine (GBM) model was built after clinicopathological variables were incorporated, with improved AUCs of 0.997 [95% CI 0.990–1.000] and 0.822 [95% CI 0.649–0.995] in the primary and validation cohort, respectively. Moreover, we constructed a combined model of CT- and MRI-based radiomic signatures that achieve better AIC (75.49 vs. 81.34 vs.82.39) than CT-based rad-score (*P* = 0.005) and MRI-based rad-score (*P* = 0.003) alone did.

**Conclusions:**

The CT-based radiomic models we constructed may provide a useful and reliable tool to predict pCR after neoadjuvant treatment, identify patients that are appropriate for a 'watch and wait' approach, and thus avoid overtreatment. Moreover, the CT-based radiomic signature may add predictive value to the MRI-based models for clinical decision making.

**Supplementary Information:**

The online version contains supplementary material available at 10.1186/s12967-021-02919-x.

## Background

Colorectal cancer is known as the third common cancer in the world, of which 70% are locally advanced rectal cancer (LARC) [1]. The current treatment for LARC (T3–4 and/or N+) is the neoadjuvant treatment followed by total mesorectal excision (TME) surgery [2]. Among the patients receiving neoadjuvant treatment, roughly 15–27% of patients can achieve pathological complete response (pCR) with no visible tumor cells in the resected tumor specimen [3]. A "watch and wait" strategy could be applied in these patients to achieve comparable survival outcomes with radical resection while avoiding surgical complications, including anastomotic leak, sexual and urinary dysfunction, and severe alteration of bowel function [3–6].

However, pCR can only be confirmed by evaluating resected specimens after surgery. Therefore, it is essential to identify patients that could achieve pCR with a reliable and non-invasive method before treatment [7]. Numerous studies have tried to develop optimal predictive panels using clinicopathological characteristics or molecular biomarkers, but they were limited by variability and insufficient sensitivity and specificity [8–11]. Imaging techniques such as computed tomography (CT), magnetic resonance imaging (MRI), and positron emission tomography are non-invasive methods that have been exploited to evaluate the therapeutic responses to neoadjuvant [12, 13]. Although they have shown promising values in response prediction, they are limited by their subjective nature and inconsistent evaluation from different radiologists [14]. Therefore, it is urgent to develop methods to better use imaging data in batch mode to stratify patients by their responsiveness to neoadjuvant treatment.

Radiomics, a fast-emerging field of image analysis, could extract high-dimension feature information from routinely acquired medical images in a high-throughput way, followed by subsequent data analysis for decision support [15, 16]. These features have been revealed to be closely associated with pathological heterogeneity [17], prognosis [18, 19], treatment response [20], and molecular phenotypes [21, 22] in tumors.

Multiple studies have recently applied radiomic analysis to predict pCR after neoadjuvant treatment in LARC patients [23–26]. However, previous CT-based models for predicting pCR after neoadjuvant treatment turn out to be controversial, which can be attributed to their retrospective design, the small size of cohorts, and non-contrast CT images that they used [27, 28]. In addition, to our best knowledge, none of previous studies has evaluated the feasibility of combing CT-based and MRI-based radiomic signatures to predict pCR.

We therefore aimed to develop a CT-based radiomic model to predict pCR by using prospectively collected imaging data in LARC patients from a randomized controlled trial (FOWARC, NCT01211210) that compared different neoadjuvant regimens [29, 30]. Moreover, we assessed the performance of an integrated model that combines CT-based and T2-weighted (T2W) MRI-based radiomic signature in pCR prediction to better guide the decision making of a "watch and wait" strategy.

## Materials and methods

### Patients

**Fig. 1:**
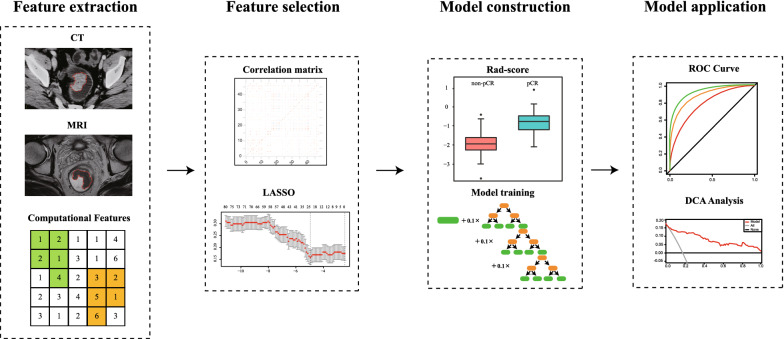
The diagram of workflow in this study

The patients enrolled in the FOWARC (NCT01211210) clinical trial [29, 30] were identified and included in this study. Briefly, all the patients were locally advanced rectal cancer (cT3–4 and/or cN1-2, c-Stage II–III), and they were randomly assigned to three neoadjuvant treatment groups as we previously described [29, 30]. Patients in each group underwent curative surgery 6–8 weeks after neoadjuvant treatment. Among these patients, 177 patients with available portal venous-phase contrast-enhanced CT image data using the same CT scanner were included in the current study. They were randomly allocated to the primary and validation cohort at a ratio of 2:1. The workflow of patient cohort disposition was shown in Fig. 1.

### Data collection and pathological response evaluation

Demographic characteristics and baseline characteristics of patients were prospectively collected or obtained from our institutional cancer database and inpatient medical records. Pathological response after neoadjuvant therapy was evaluated using the tumor response grading (TRG) system [31] by two pathologists in consensus. Patients were divided into two different response groups: pCR group (TRG 0, no viable residual tumor cells) and non-pCR group (TRG 1–4, varying from rare residual cancer cells to extensive residual cancer cells).

### Image data acquisition and tumor segmentation

All patients underwent CT scans within 1 week before neoadjuvant treatment. The imaging data were retrieved from the picture archiving and communication system (PACS, Carestream, Canada). The details of the imaging protocol were provide in Additional file 1. As shown in Additional file 1: Figure S1, the region of interest (ROI) covering the whole tumor was manually outlined along the margin of tumors by two experienced radiologists using the itk-SNAP software (version 3.8.0, www.itksnap.org). The robustness of each ROI outlining and inter-/intra-observer reproducibility was evaluated by calculating the intra- and inter-class correlation coefficients (ICCs). Both of the radiologists were blinded to the clinicopathological information of each case.

### Radiomic features extraction

The radiomic features were preprocessed and extracted by Pyradiomics (Version 2.1.2) as previously described [32]. Two methods of filters were applied to preprocess CT images: Laplacian of Gaussian (an edge enhancement filter that emphasizes areas of gray level change) [33] and Wavelet filtering (a filter yielding eight decompositions per level in each of the three dimensions) [34]. Each original image was normalized with z-score before being processed by filters. A total of 1218 radiomic features were then acquired from CT images in each patient, including the first-order statistics and other statistics derived from the Gray-Level Co-occurrence Matrix (GLCM), Gray Level Run Length Matrix (GLRLM), Gray Level Size Zone Matrix (GLSZM), and Gray Level Dependence Matrix (GLDM) [35]. More details about the feature extraction procedure and parameter settings could be found in Additional file 1.

### Feature selection and radiomic signature construction

We took multiple steps to identify the pCR-associated radiomic features (Additional file 1). First, we evaluated the overall pair-wise correlation and excluded highly correlated features based on the cut-off value (*ρ* = 0.85) to select candidate features for further analysis [24, 36]. Second, a logistic regression model optimized by the least absolute shrinkage and selection operator (LASSO) method was applied to further select representative features that were associated with achieving pCR [37]. Finally, the radiomic signature, termed as rad-score, of each patient was calculated through a linear combination of estimated coefficients and radiomic values of each selected feature.

### Development and validation of predicting models

We applied three different models to integrate rad-score with clinicopathological predictors, including logistic regression, support vector machine (SVM) [38] and gradient boosting machine (GBM) [39]. Each model was trained and tuned (if needed) by a five-times ten-fold repeated cross-validation. More information about the models was provided in Additional file 1: Table S3.

### Development of a model integrating CT- and MRI-based radiomic features

The pre-treatment T2W MRI images of 99 patients were retrieved from PACS. The tumor segmentation and radiomic signature construction of MRI images were performed as they were done in CT image analyses. The multivariate logistic regression analysis was used to integrate the CT-based and MRI-based rad-scores for pCR prediction. The comparison between the CT-based, MRI-based and CT-MRI rad-score was assessed using likelihood ratio (LR) and Akaike information criterion (AIC). In general, the model with a lower AIC was considered a better one.

### Statistical analysis

Demographic and clinicopathological characteristics were compared between the pCR and non-pCR groups using *t*-test or Mann–Whitney U tests (for continuous numerical variables depending on their distributions) and chi-squared tests or two-tailed Fisher's exact tests (for categorical variables). All the statistical analyses were conducted with R software version 3.6.2 (http://www.R-project.org). The model construction, parameter tuning, comparison and assessment were performed using the "caret" package. The Receiver operating characteristic (ROC) analyses were conducted by using the "pROC" package. Model performance was assessed with the average area-under-receiver-operator-curve (AUC), accuracy, specificity, and sensitivity. A decision curve analysis (DCA) of the logistic regression model was conducted to evaluate the clinical practicability by calculating the net benefits at different threshold probabilities. The statistical significance levels were all set to be 0.05 with two sides.

## Results

### Demographic and clinicopathological characteristics

The baseline characteristics of patients in the primary cohort and validation cohort were shown in Table 1. There were 113 and 64 patients in the primary and validation cohort, respectively. Among them, 20 (17.70%) and 11 patients (17.19%) achieved pCR in the primary and validation cohort, respectively. The tumor thickness was significantly different between pCR and non-pCR patients in the primary cohort but not in the validation cohort. There were no significant differences in other clinicopathological characteristics between pCR and non-pCR patients in either cohort.

**Table 1:** Clinical characteristics of patients in the primary and validation cohorts

Characteristics	Training cohort		*p*	Validation cohort		*p*
	Non-pCR (N = 93)	pCR (N = 20)		Non-pCR (N = 53)	pCR (N = 11)	
Age (yr)			.569			.847
≤ 60	62 (66.7%)	12 (60.0%)		37 (69.8%)	8 (72.7%)	
> 60	31 (33.3%)	8 (40.0%)		16 (30.2%)	3 (27.3%)	
Gender			.668			.586
Female	28 (30.1%)	7 (35.0%)		19 (35.8%)	3 (27.3%)	
Male	65 (69.9%)	13 (65.0%)		34 (64.2%)	8 (72.7%)	
cT stage			.494			.847
III	80 (86.0%)	16 (80.0%)		42 (79.2%)	9 (81.8%)	
IV	13 (14.0%)	4 (20.0%)		11 (20.8%)	2 (18.2%)	
cN stage			.197			.707
0	27 (29.0%)	3 (15.0%)		7 (13.2%)	1 (9.1%)	
1	66 (71.0%)	17 (85.0%)		46 (86.8%)	10 (90.9%)	
cTNM stage			.411			.512
II	27 (29.0%)	4 (20.0%)		9 (17.0%)	1 (9.1%)	
III	66 (71.0%)	16 (80.0%)		44 (83.0%)	10 (90.9%)	
MRF			.082			.177
Negative	73 (78.5%)	12 (60.0%)		32 (60.4%)	9 (81.8%)	
Positive	20 (21.5%)	8 (40.0%)		21 (39.6%)	2 (18.2%)	
TL (cm)			.625			.266
≤ 3	23 (24.7%)	6 (30.0%)		11 (20.8%)	4 (36.4%)	
> 3	70 (75.3%)	14 (70.0%)		42 (79.2%)	7 (63.6%)	
DTVA (cm)			.351			.549
≤ 5	36 (38.7%)	10 (50.0%)		19 (35.8%)	5 (45.5%)	
> 5	57 (61.3%)	10 (50.0%)		34 (64.2%)	6 (54.5%)	
CEA (ng/mL)			.235			.214
≤ 5	64 (68.8%)	11 (55.0%)		33 (62.3%)	9 (81.8%)	
> 5	29 (31.2%)	9 (45.0%)		20 (37.7%)	2 (18.2%)	
Tumor thickness (mm) Median (Q1, Q3)	14.00 (11.00, 17.00)	15.50 (13.00, 21.25)	.029*	16.00 (12.00, 18.00)	13.00 (12.50, 18.00)	.419
Rad-score Median (Q1, Q3)	− 1.89 (− 2.21, − 1.56)	− 0.71 (− 1.15, − 0.42)	< .001*	− 1.95 (− 2.28, − 1.51)	− 0.54 (− 1.17, − 0.29)	**<** .001*

pCR: Complete pathological response; MRF: Mesorectal fascia; TL: Tumor length; DTVA: Distance of tumor from the anal verge; CEA: Carcinoembryonic antigen; cT stage: Clinical T stage, cN stage: Clinical N stage

### Feature selection and radiomic signature construction for predicting pCR

**Fig. 2:**
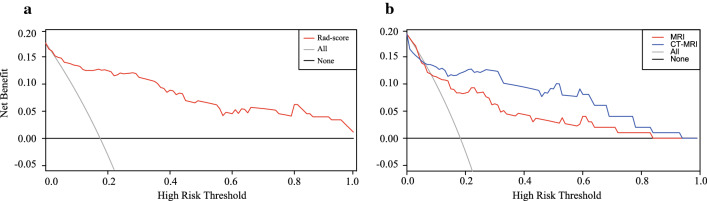
The decision curve analysis in this study. The decision curve analysis showed that using the CT-based rad-score to predict pCR added benefit than treating either all or no patients did when the threshold probability was between 0 and 1 (**A**) and using the CT-MRI-based integrated model gained more benefit when comparing with the MRI-based rad-score (**B**). The y-axis measured the net benefit. The x-axis represented the threshold probability. The red line represented the radiomic model. The grey line represented the assumption that all patients achieved pCR. The black line represented the hypothesis that no patients achieved pCR

**Fig. 3:**
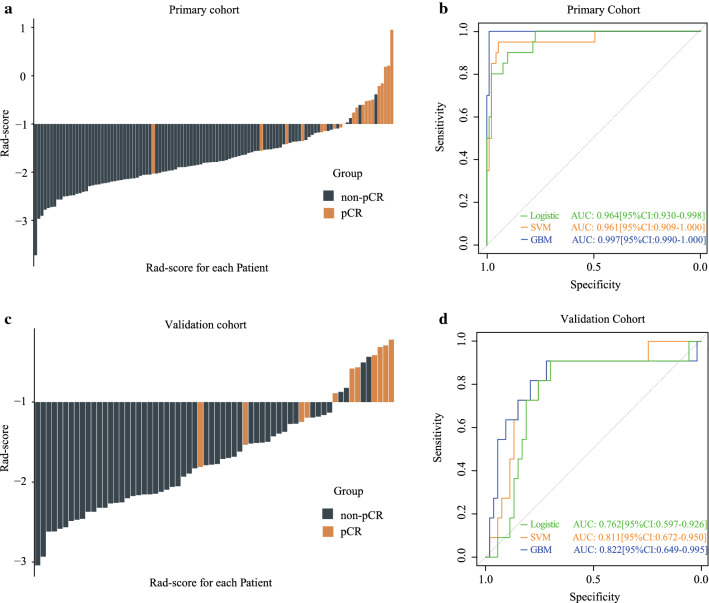
Performance of the multivariable radiomic models. The CT-based rad‐score for each patient in the primary cohort (**A**) and the validation cohort (**C**), respectively; The ROC curves of the CT-based radiomic models using different methods in the primary cohort (**B**) and the validation cohort (**D**), respectively

At the first stage of feature selection, we followed the criterion that features with relatively high overall pair-wise correlation would be removed. Accordingly, 272 selected features were then included into the LASSO regression model and 14 features were finally selected to build the rad-score (Additional file 1). The selected features contained 4 first-order features, 4 GLSZM features, 3 GLCM features, 2 GLDM features, and 1 shape feature from 13 different filtrations. Detailed information of the selected features was provided in Additional file 1: Table S1 and Table S2. The rad-scores of the pCR group were significantly higher than those in the non-pCR group in both the primary (*P* < 0.001) and validation (*P* < 0.001) cohort. Of note, the distributions of rad-scores in both cohorts were shown in Fig. 3A and C, in which the majority of patients achieving pCR had a high rad-score in both cohorts. The decision curve analysis (DCA) for the CT-based rad-score confirmed its application in clinical decision making (Fig. 2A).

### Development and validation of integrated models for predicting pCR

We further constructed models that integrated rad-score with clinicopathological predictors to better predict pCR. The multivariate logistic regression, SVM, and GBM models were constructed as shown in Additional file 1: Fig.S2. The top-ranked predictive variables were rad-score, CEA, MRF, and tumor thickness in the GBM model. Detailed information of these models was given in Additional file 1: Fig. S3.

The SVM model and GBM model had better predictive performance than the logistic model (Fig. 3B and D). The SVM model had an AUC of 0.961 [95% CI, 0.909–1.000] and 0.811 [95% CI, 0.672–0.950] in the primary and validation cohort, respectively, while the GBM model yielded the highest AUCs of 0.997 [95% CI 0.990–1.000] in the primary cohort and 0.822 [95% CI, 0.649–0.995] in the validation cohort. Moreover, the GBM model had the best accuracies, sensitivities, and specificities in both cohorts (Table 2). Taken together, the GBM model was selected for further analysis with the MRI-based radiomic model.

**Table 2:** Performances of the radiomics models in the primary cohort and validation cohort

	Accuracy (95% CI)	Sensitivity (95% CI)	Specificity (95% CI)
Primary cohort			
Logistic	0.903 (0.833–0.950)	0.900 (0.669–0.982)	0.903 (0.820–0.952)
SVM	0.912 (0.843–0.957)	0.950 (0.731–0.997)	0.903 (0.820–0.952)
GBM	0.973 (0.924–0.995)	1.000 (0.799–1.000)	0.946 (0.902–0.992)
Validation cohort			
Logistic	0.797 (0.678–0.887)	0.727 (0.393–0.927)	0.811 (0.676–0.901)
SVM	0.766 (0.643–0.863)	0.818 (0.478–0.968)	0.755 (0.614–0.858)
GBM	0.813 (0.695–0.899)	0.727 (0.393–0.927)	0.830 (0.697–0.915)

SVM: support vector machine; GBM: gradient boosting machine

### CT-based radiomic signature contributes to MRI-based radiomic model

To explore the values that CT-based radiomic signature could add to previously reported MRI-based radiomic models, we retrieved the pre-treatment T2W MRI images of 99 patients from the PACS system and performed radiomic analyses. After feature extraction, a total of 21 MRI features were selected and the MRI-based rad-score was constructed. CT-MRI rad-scores were calculated for each patient in this subset (Additional file 1). The multivariate logistic regression analysis showed that both the CT-based rad-score (*P* = 0.010) and MRI-based rad-score (*P* = 0.011) were independent predictive factor (Additional file 1: Table S4). As shown in Table 3, the performance of the integrated model (CT-MRI rad-score) was significantly better than CT (*P* = 0.005) or MRI (*P* = 0.003) alone did (AIC: 75.49 vs. 81.34 vs. 82.39). The DCA curves also showed that the integrated model performed better for predicting pCR in this subset (Fig. 2B).

**Table 3:** Model fit among three models

Models	AIC	Brier score	*P*
CT-based rad-score	81.34	0.099	0.005^a^
MRI-based rad-score	82.39	0.120	0.003^b^
CT-MRI rad-score	75.49	0.087	

AIC, Akaike information criterion value

^a,b^*p* value for the Likelihood ratio test in CT-based and MRI-based rad-scores compared with CT-MRI rad-score

## Discussion

In this post-hoc analysis derived from a prospectively randomized controlled trial, we initially used machine learning methods to construct a pCR-associated rad-score based on radiomic features extracted from pre-treatment portal venous-phase contrast-enhanced CT images. The CT-based rad-scores were significantly different between pCR and non-pCR patients in the primary and validation cohort, respectively. We next integrated the rad-score with clinicopathological variables to develop multiple predictive models for pCR. Among the models, the GBM model had the best performance with AUCs of 0.997 and 0.822 in the primary and validation cohort. Moreover, we integrated the CT-based and MRI-based radiomic signatures to construct an improved model for pCR prediction with a better AUC compared to CT or MRI alone. The models we constructed may provide a useful and reliable tool to identify pCR patients that are appropriate for a 'watch and wait' approach.

A total of 1218 candidate radiomic features were extracted from the primary tumor region. The first order features including Median and Kurtosis accounted for a large proportion in our radiomic signature (4/14, Additional file 1: Table S1). Previous texture analysis based on CT [40] and MRI [41] have also reported the importance of first order features in predicting treatment response and prognosis. These features basically evaluated voxel intensities, which reflect the shape and volume irregularity of tumors, which are difficult to be identified with naked eyes. The Gray Level Size Zone Matrix (GLSZM) features, which quantify gray level zones (defined as the number of connected voxels that share the same gray level intensity) in an image, contributed to our radiomic signatures as well (4/14, Additional file 1: Table S1), It has been shown that tumors with higher heterogeneity tend to get aberrant values in these radiomic features and our result is consistent with the hypothesis that radiomic analysis may reflect tumor heterogeneity associated with underlying molecular phenotypes [42, 43]. Comparing with other studies that applied the features merely from the primary images [28, 44] we used Laplacian of Gaussian (LoG) and Wavelet filters to preprocess the images into multiple filtered images through different scales of smoothing [45]. Preprocessing images with filters can enhance the high-dimensional features of tumors that are difficult to be recognized in direct visual assessment by reducing the hybrid texture of tissue adjacent to tumor and previous studies have promoted their radiomic models using these filters [46]. The enrolled LoG and Wavelet features (13/14, Additional file 1: Table S1) in our study also validated that image filtering before feature extraction may identify more valuable radiomic features for outcome prediction and improve the performance of constructed models.

Several studies tried to construct predicting models for pCR based on clinicopathological characteristics. In our study, we also combined available clinical characteristics with rad-scores by a GBM method and the clinical variables that contribute to the model include CEA, tumor thickness, MRF, clinical N stage, which is consistent with previous studies based on a large sample size [8–11, 47, 48]. Decision trees have shown good performance with the application of this model, which is recently applied to statistical learning methods for classification and regression. However, our result showed that the clinical variables contribute less to pCR prediction compared with the CT-based rad-scores. It could be anticipated that incorporating variables with more dimensions such as molecular biomarkers may further improve current models.

Some recent studies have constructed radiomic models to identify patients that may achieve pCR after neoadjuvant treatment. Among them, most investigated the predictive value of radiomic features for pCR based on multiparametric MRI, and the AUCs of these models were reported to be 0.712–0.966 [25, 36, 38]. The analyzed MRI data included T1W [36], T2W [25] and DWI [24] images from pre-treatment to post-treatment [44]. However, few of them focused on CT-based radiomics analysis, although it has been demonstrated that multiple radiomics analysis based on CT images can facilitate the prediction of lymph node metastasis [37, 49, 50], distant metastasis [51], therapy response [52, 53] and prognostic outcomes [28]. Two previous studies have performed CT-based radiomics analysis for pCR prediction but came out with controversial results [27, 54]. Both of these studies analyzed non-contrast CT images, which may not display tumor characteristics well, and were based on small sizes of cohorts with retrospective design [27, 55]. Comparing to these studies, one advantage of our study is that we better controlled the imbalanced distribution of confounding factors by enrolling patients from a randomized controlled trial. Moreover, different from some studies based on non-contrast CT [27], we used the portal venous-phase contrast-enhanced CT images that are commonly approved to be more informative in interpreting tumor tissues. With these advantages, our CT-based model had an AUC as high as 0.997 and 0.822 in the primary and validation cohort, which seemed to be superior to MRI-based models in previous studies [25, 36, 38]. Our results add reliable evidence for pre-treatment CT-based radiomics analysis in predicting pCR after neoadjuvant treatment. It is well known that MRI and CT may interpret tumor characteristics in different physiological and biological modalities [56, 57]. Previous studies have constructed predictive models based on CT, MRI or their combined signature for tumor progression in various cancers [58, 59]. Their results proved that CT and MRI have unique contributions in predicting outcomes. Based on this context, we innovatively explored the additional benefits of CT-based radiomic signatures for previously reported MRI-based models and constructed a novel integrated model. Expectedly, a significant improvement was determined in the integrated rad-score with better AIC and brier score compared to either of them alone. To the best of our knowledge, our study is the first to combine CT and MRI images together to perform radiomics analysis in rectal cancer, especially in pCR prediction, and the result showed that it might interpret rectal tumors more comprehensively. This novel modality of predicting model deserves to be further investigated and validated in a large cohort.

As the "watch and wait" strategy is recently proposed to be an alternative strategy for patients with clinical complete response (cCR), it is urgent to have a reliable and accurate method to distinguish pCR before surgery and guide clinical decision making among these patients. Considering the predictive value of our radiomic models constructed before treatment for pCR, they could at least assist clinicians in distinguishing cCR patients achieving pCR from those not achieving pCR after neoadjuvant treatment. The decision curve analysis (DCA) demonstrated that the radiomic signatures might add more net benefit in the clinical practice than the 'treat all' or 'treat none' strategies. This model could be a quantitative and reliable tool in deciding which patients need radical surgery and which patients are suitable for the "watch and wait" strategy.

The robustness of this study mainly came from the prospective patient cohort and homogeneity of imaging data that were applied in radiomics analysis, though there are some limitations in our study. First, the sample size of patients with pCR was small in our cohorts, which may introduce bias and bring the inaccuracy and instability to the predictive models. Second, this was a secondary study of a clinical trial. Validation of the proposed models in an independent cohort is warranted in further study before clinical application. Third, the integrated model that enrolled CT-based and MRI-based radiomic signatures is an exploratory and preliminary test with a limited sample size. The additional studies for model optimization and validation in patient cohorts would be necessary.

## Conclusion

This post-hoc study of a randomized controlled trial developed and validated a pre-treatment enhanced CT-based rad-score to accurately predict pCR after neoadjuvant treatment in LARC patients. We further integrated the rad-score with clinicopathological variables to develop a GBM model for pCR prediction with improved performance. Moreover, we explored the predictive value CT-based radiomic signature could add to the MRI-based models that were reported previously and proposed a novel comprehensive model that performed better than CT or MRI alone. These models could be useful tools to help clinical decision making in rectal cancer patients receiving neoadjuvant treatment.

## Supplementary Information

**Additional file 1.** Additional material about the additional descriptions of the imaging protocol, radiomic features and model building.

## Data Availability

The datasets used during the current study are available from the corresponding author on reasonable request.
