# Supplementary material for: Radiomic signature of the FOWARC trial predicts pathological response to neoadjuvant treatment in rectal cancer
Source: J Transl Med. 2021 Jun 10;19:256. doi: 10.1186/s12967-021-02919-x (PMC8194221; doi:10.1186/s12967-021-02919-x)
Supplement: Supplementary file 1 — Additional file 1. Additional material about the additional descriptions of the imaging protocol, radiomic features and model building. [file 12967_2021_2919_MOESM1_ESM.docx]

**Supplementary material**

**Supplementary Methods1: Imaging protocol and parameter setting**

**1. CT**

All CT scans were performed by one model machine (GE Optima CT660). Each patient underwent a contrast enhanced CT scan from the neck to the abdomen. After routine non-enhanced CT, 1 to 1.5 ml/kg of the contrast agent iopromide 370 was injected into the vein at a rate of 2.5 to 4 ml/s by using the BAB injection method. After 20s to 30s, the arterial phase contrast enhanced CT was implemented.

The technical CT parameters are listed as follow: Kilovolt peak (KVP): 120 kV; Effective mAs: Auto mA; Rotation Time: 0.5s or 0.6s; Detector Collimation: 64 × 0.625 mm or 80 × 0.5 mm; Reconstruction Slice Thickness: 1.25 mm or 1 mm; Field of View: 250 × 250 mm2 or 220 × 220 mm2 (matrix: 512 × 512 pixels).

**2. MRI**

All patients were scanned with 1.5 Tesla MR (Optima MR 360, GE Medical Systems, USA) using an eight-element body array coil with fixed image protocols. Patients did not receive any special bowel preparation before the MR examination. Each patient underwent a series of 4 MRI scanning sequences, including DWI, T1w, CE-T1w, and T2w.

The technical MRI parameters of T2w are listed as follow: TR/TE: 4800-5000/102 msec, Slice thickness 3.0mm, Spacing 0.5mm, Echo Train Length 24; Frequency 288; Phase 256; NEX 4.0, Bandwidth 31.255 kHz.


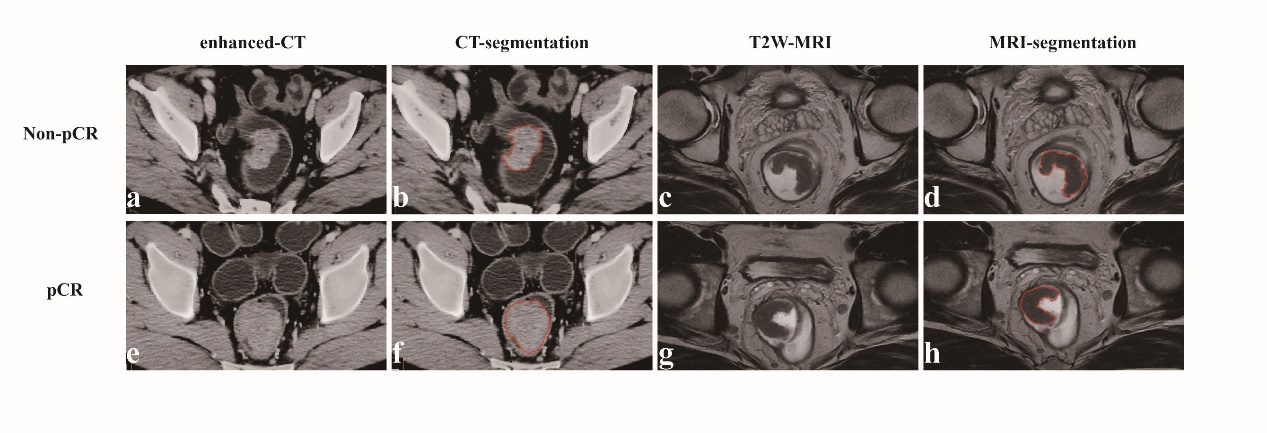


**Figure**.**S1 Tumor segmentation.** Four cases of tumor segmentation in portal venous-phase contrast-enhanced CT images and T2-weighted-MRI images. Two patients achieving pCR and two non-pCR patients were demonstrated. Segmentation of ROI for the four cases were shown

**Supplementary Methods2: Details of image filtration and radiomics features**

**1. Image filtration**

**(1) Laplacian of Gaussian (LoG)**

A Laplacian of Gaussian image is obtained by convolving the image with the second derivative (Laplacian) of a Gaussian kernel. A low sigma emphasis on fine textures (change over a short distance), where a high sigma value emphasises coarse textures (gray level change over a large distance). In this study, 1.0, 2.0, 3.0, 4.0, 5.0 were selected as *σ*, respectively and five LoG-processed images were obtained.

**(2) Wavelet**

In this study, eight wavelet-processed images were obtained as HHH, HHL, HLH, HLL, LHH, LHL, LLH, LLL.

**2. Radiomics features**

**(1)** **First Order Features**

1. Energy

2. Total Energy

3. Entropy

4. Minimum

5. 10th percentile

6. 90th percentile

7. Maximum

8. Mean

9. Median

10. Interquartile Range

11. Range

12. Mean Absolute Deviation (MAD)

13. Robust Mean Absolute Deviation (rMAD)

14. Root Mean Squared (RMS)

15. Skewness

16. Kurtosis

17. Variance

18. Uniformity

**(2)** **Gray Level Co-occurrence Matrix (GLCM) Features**

1. Autocorrelation

2. Joint Average

3. Cluster Prominence

4. Cluster Shade

5. Cluster Tendency

6. Contrast

7. Correlation

8. Difference Average

9. Difference Entropy

10. Difference Variance

11. Joint Energy

12. Joint Entropy

13. Informational Measure of Correlation (IMC)1

14. Informational Measure of Correlation (IMC)2

15. Inverse Difference Moment (IDM)

16. Inverse Difference Moment Normalized (IDMN)

17. Inverse Difference (ID)

18. Inverse Difference Normalized (IDN)

19. Inverse Variance

20. Maximum Probability

21. Sum Entropy

22. Sum of Squares

**(3)** **Gray Level Dependence Matrix (GLDM) Features**

1. Small Dependence Emphasis (SDE)

2. Large Dependence Emphasis (LDE)

3. Gray Level Non-Uniformity (GLN)

4. Dependence Non-Uniformity (DN)

5. Dependence Non-Uniformity Normalized (DNN)

6. Gray Level Variance (GLV)

7. Dependence Variance (DV)

8. Dependence Entropy (DE)

9. Low Gray Level Emphasis (LGLE)

10. High Gray Level Emphasis (HGLE)

11. Small Dependence Low Gray Level Emphasis (SDLGLE)

12. Small Dependence High Gray Level Emphasis (SDHGLE)

13. Large Dependence Low Gray Level Emphasis (LDLGLE)

14. Large Dependence High Gray Level Emphasis (LDHGLE)

**(4) Gray Level Run Length Matrix (GLRLM) Features**

1. Short Run Emphasis (SRE)

2. Long Run Emphasis (LRE)

3. Gray Level Non-Uniformity (GLN)

4. Gray Level Non-Uniformity Normalized (GLNN)

5. Run Length Non-Uniformity (RLN)

6. Run Length Non-Uniformity Normalized (RLNN)

7. Run Percentage (RP)

8. Gray Level Variance (GLV)

9. Run Variance (RV)

10. Run Entropy (RE)

11. Low Gray Level Run Emphasis (LGLRE)

12. High Gray Level Run Emphasis (HGLRE)

13. Short Run Low Gray Level Emphasis (SRLGLE)

14. Short Run High Gray Level Emphasis (SRHGLE)

15. Long Run Low Gray Level Emphasis (LRLGLE)

16. Long Run High Gray Level Emphasis (LRHGLE

**(5) Gray Level Size Zone Matrix (GLSZM) Features**

1. Small Area Emphasis (SAE)

2. Large Area Emphasis (LAE)

3. Gray Level Non-Uniformity (GLN)

4. Gray Level Non-Uniformity Normalized (GLNN)

5. Size-Zone Non-Uniformity (SZN)

6. Size-Zone Non-Uniformity Normalized (SZNN)

7. Zone Percentage (ZP)

8. Gray Level Variance (GLV)

9. Zone Variance (ZV)

10. Zone Entropy (ZE)

11. Low Gray Level Zone Emphasis (LGLZE)

12. High Gray Level Zone Emphasis (HGLZE)

13. Small Area Low Gray Level Emphasis (SALGLE)

14. Small Area High Gray Level Emphasis (SAHGLE)

15. Large Area Low Gray Level Emphasis (LALGLE)

16. Large Area High Gray Level Emphasis (LAHGLE)

**(6) Shape Features (only in original images)**

1. Elongation

2. Least Axis Length

3. Major Axis Length

4. Maximum 2D diameter (Column)

5. Maximum 2D diameter (Row)

6. Maximum 2D diameter (Slice)

7. Maximum 3D diameter

8. Mesh Volume

9. Minor Axis Length

10. Sphericity

11. Surface Area

12. Flatness

13. Surface Area to Volume ratio

14. Voxel Volume


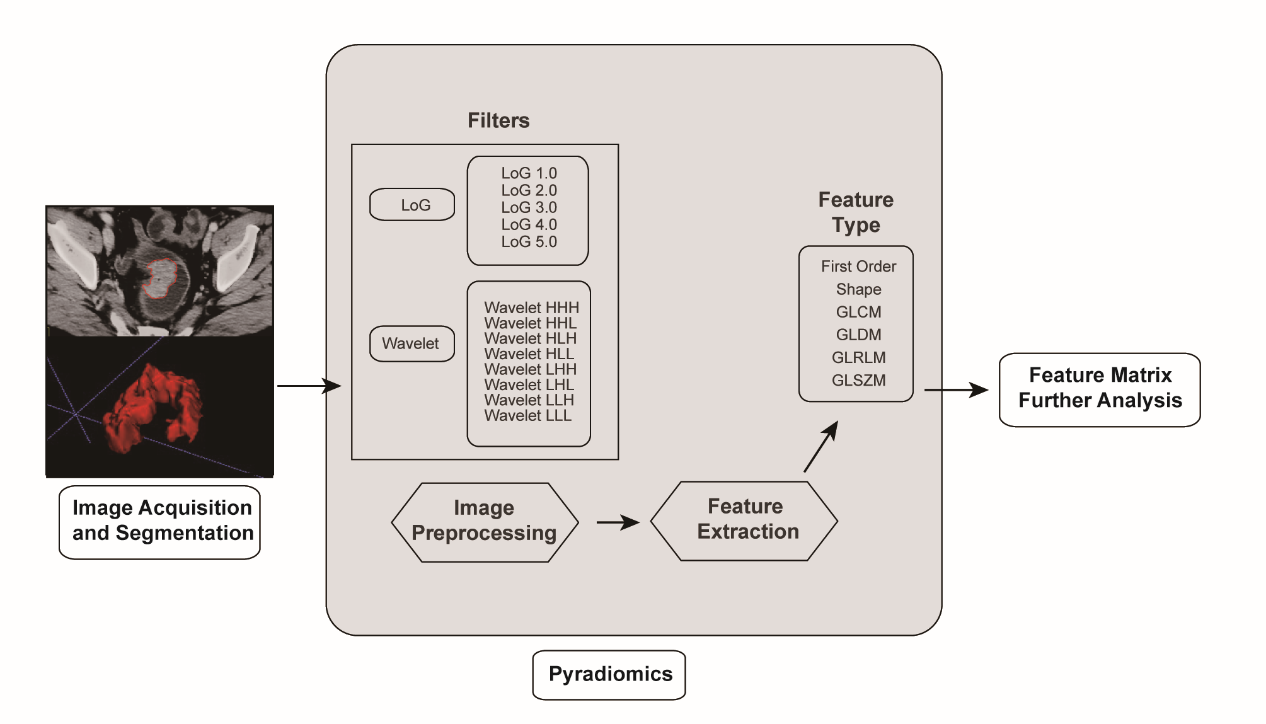


**Figure**.**S2 The process of feature extraction using Pyradiomics platform.**

**Supplementary Methods3: Feature Selection**

**1. Feature Correlation Matrix**

It is possible that the originally extracted features might contain overlapped information, which would increase the model complexity and result in a low efficiency. For the 1218 extracted CT-based features, a correlation matrix was firstly constructed to identify the highly correlated features. By evaluating the overall pair-wise Spearman correlation coefficients presented in the matrix, we excluded highly correlated features by setting the cut-off value of correlation coefficient *ρ* as 0.85. For the 1132 MRI-based features, a similar correlation matrix (*ρ* = 0.85) was constructed with the same purpose. **Table**.**S1** shows the distribution of selected CT-based and MRI-based radiomics features of different image filter types.

**Table**.**S1** The distribution of selected CT-based and MRI-based radiomics features in different filters.

| **image type** | **feature** | **coefficient** | **filter** |
| --- | --- | --- | --- |
| **CT** | **(Intercept)** | -1.689 | - |
|  | **firstorder_Median** | -0.001 | wavelet. HHL |
|  | **firstorder_Median** | -0.168 | wavelet. HLH |
|  | **firstorder_Kurtosis** | -0.207 | wavelet. LHL |
|  | **firstorder_Kurtosis** | -0.073 | wavelet. LLH |
|  | **glcm_InverseVariance** | -0.109 | wavelet. LHL |
|  | **glcm_Imc2** | -0.059 | wavelet. LLL |
|  | **glcm_Idn** | 0.010 | LoG 5.0 |
|  | **gldm_DNN** | 0.195 | wavelet. HLL |
|  | **gldm_DV** | -0.038 | LoG 4.0 |
|  | **glszm_SALGLE** | -0.458 | wavelet. HLH |
|  | **glszm_ SALGLE** | -0.145 | LoG 4.0 |
|  | **glszm_ LALGLE** | 0.195 | LoG 5.0 |
|  | **glszm_ SZNN** | -0.036 | wavelet. HLH |
|  | **shape_Maximum3DDiameter** | 0.034 | original |
| **MRI** | **(Intercept)** | -1.893 |  |
|  | **glcm_Idmn** | 0.394 | LoG 2.0 |
|  | **gldm_LDE** | -0.659 | LoG 3.0 |
|  | **glszm_SZNUN** | 0.605 | LoG 3.0 |
|  | **firstorder_RootMeanSquared** | -0.016 | LoG 4.0 |
|  | **glszm_SAHGLE** | -0.601 | LoG 5.0 |
|  | **firstorder_Kurtosis** | 0.086 | wavelet. HHH |
|  | **glcm_Contrast** | -0.097 | wavelet. HHL |
|  | **gldm_SDHGLE** | -0.264 | wavelet. HHL |
|  | **glszm_SZNU** | 0.401 | wavelet. HHL |
|  | **glcm_Idn** | 0.735 | wavelet. HLH |
|  | **firstorder_Kurtosis** | -0.348 | wavelet. HLL |
|  | **firstorder_Maximum** | -0.792 | wavelet. HLL |
|  | **firstorder_RootMeanSquared** | -0.117 | wavelet. HLL |
|  | **firstorder_Kurtosis** | -0.198 | wavelet. LHH |
|  | **firstorder_RootMeanSquared** | 0.055 | wavelet. LHH |
|  | **firstorder_RootMeanSquared** | -0.111 | wavelet. LHL |
|  | **glcm_Idn** | 0.370 | wavelet. LLH |
|  | **glrlm_SRHGLE** | 0.319 | wavelet. LLH |
|  | **gldm_LDLGLE** | 0.125 | wavelet. LLL |
|  | **shape_Flatness** | 0.388 | original |
|  | **shape_MinorAxisLength** | -0.412 | original |

**2. Logistic LASSO Regression**

Two Lasso logistic models were constructed for selecting CT and MRI features, respectively. The models finally kept 14 CT features and 12 MRI features, which were then used to calculate the CT-based rad-score and MRI-based rad-score. **Table**.**S2** contained the tuning parameters for the LASSO models obtained via a ten-fold cross-validation. The coefficients obtained from the LASSO models were used to calculate the CT-based rad-score and MRI-based rad-score, respectively. Values of the selected features were normalized before linearly combined with the coefficients. The CT-based rad-score has been calculated for all patients (n=177) and the MRI-score has been calculated for the patients of a subcohort (n=99) while adjusting the prevalence of response.

| **Table.S2 Parameters of the LASSO Models** | | | |
| --- | --- | --- | --- |
| **Image Type** | **Number of Features** | **Number of Selected Features** | **Regularization Parameter** |
| **CT** | 272 | 14 | 0.054 |
| **MRI** | 158 | 21 | 0.012 |

**3. Radiomics score calculation formula**

(1) CT-based Rad-score = -1.689-0.001×wavelet.HHL_firstorder_Median-0.168×wavelet.HLH_firstorder_Median-0.207×wavelet.LHL_firstorder_Kurtosis-0.073×wavelet.LLH_firstorder_Kurtosis-0.109×wavelet.LHL_glcm_InverseVariance-0.059×wavelet.LLL_glcm_Imc2+0.010×LoG5.0_glcm_Idn+0.195×wavelet.HLL_gldm_DNN-0.038×LoG4.0_gldm_DV-0.458×wavelet.HLH_glszm_SALGLE-0.145×LoG4.0_glszm_SALGLE+0.195×LoG5.0_glszm_LALGLE-0.036×wavelet.HLH_glszm_SZNN+0.034×original_shape_Maximum3DDiameter

(2) MRI-based Rad-score = -1.893+0.394×LoG2.0_glcm_Idmn-0.659×LoG3.0_gldm_LDE+0.605×LoG3.0_glszm_SZNUN-0.016×LoG4.0_firstorder_RootMeanSquared-0.601×LoG5.0_glszm_SAHGLE+0.086×wavelet.HHH_firstorder_Kurtosis-0.097×wavelet.HHL_glcm_Contrast-0.264×wavelet.HHL_gldm_SDHGLE+0.401×wavelet.HHL_glszm_SZNU+0.735×wavelet.HLH_glcm_Idn-0.348×wavelet.HLL_firstorder_Kurtosis-0.792 ×wavelet.HLL_firstorder_Maximum-0.117×wavelet.HLL_firstorder_RootMeanSquared-0.198×wavelet.LHH_firstorder_Kurtosis+0.055×wavelet.LHH_firstorder_RootMeanSquared-0.111×wavelet.LHL_firstorder_RootMeanSquared+0.37×wavelet.LLH_glcm_Idn+0.319×wavelet.LLH_glrlm_SRHGLE+0.125×wavelet.LLL_gldm_LDLGLE+0.388×original_shape_Flatness-0.412×original_shape_MinorAxisLength

**Supplementary Methods4: Model Construction**

To construct predicting models, we enrolled 9 variables as clinicopathological predictors: age, gender, cT stage, cN stage, MRF status, tumor length, DTVA (Distance of tumor from the anal verge), CEA, tumor thickness. Demographic characteristics and baseline characteristics of patients were prospectively collected or obtained from institutional cancer database and inpatient medical records. Tumor-related parameters such as cT, cN, MRF status, DTAV, tumor length and tumor thickness were measured using MRI and/or colonoscopy. Tumor length was defined as the maximum diameter of tumor. Tumor thickness was defined as the deepest point of the rectal wall invasion.

**Table.S3. Parameter Information of predicting models**

| **Model** | **number of predictors** | **Tuning Parameters** | **Best Tune** | **Grid Search Range** | **Metric** |
| --- | --- | --- | --- | --- | --- |
| **Logistic** | 8 | - | - | - | AUC |
| **SVM** | 11 | cost | 3.74 | 1~5 |  |
| **GBM of the CT-based model** | 11 | number of trees | 2000 | 2000, 2500 |  |
|  |  | max tree depth | 1 | 1 |  |
|  |  | shrinkage | 0.02 | 0.02 |  |
|  |  | minimal node size | 1 | 1 |  |
| **GBM of the integrated model** | 11 | number of trees | 2000 | 2000, 2500, 3000 |  |
|  |  | max tree depth | 1 | 1~4 |  |
|  |  | shrinkage | 0.0015 | 0.001, 0.0015, 0.002 |  |
|  |  | minimal node size | 2 | 2 |  |


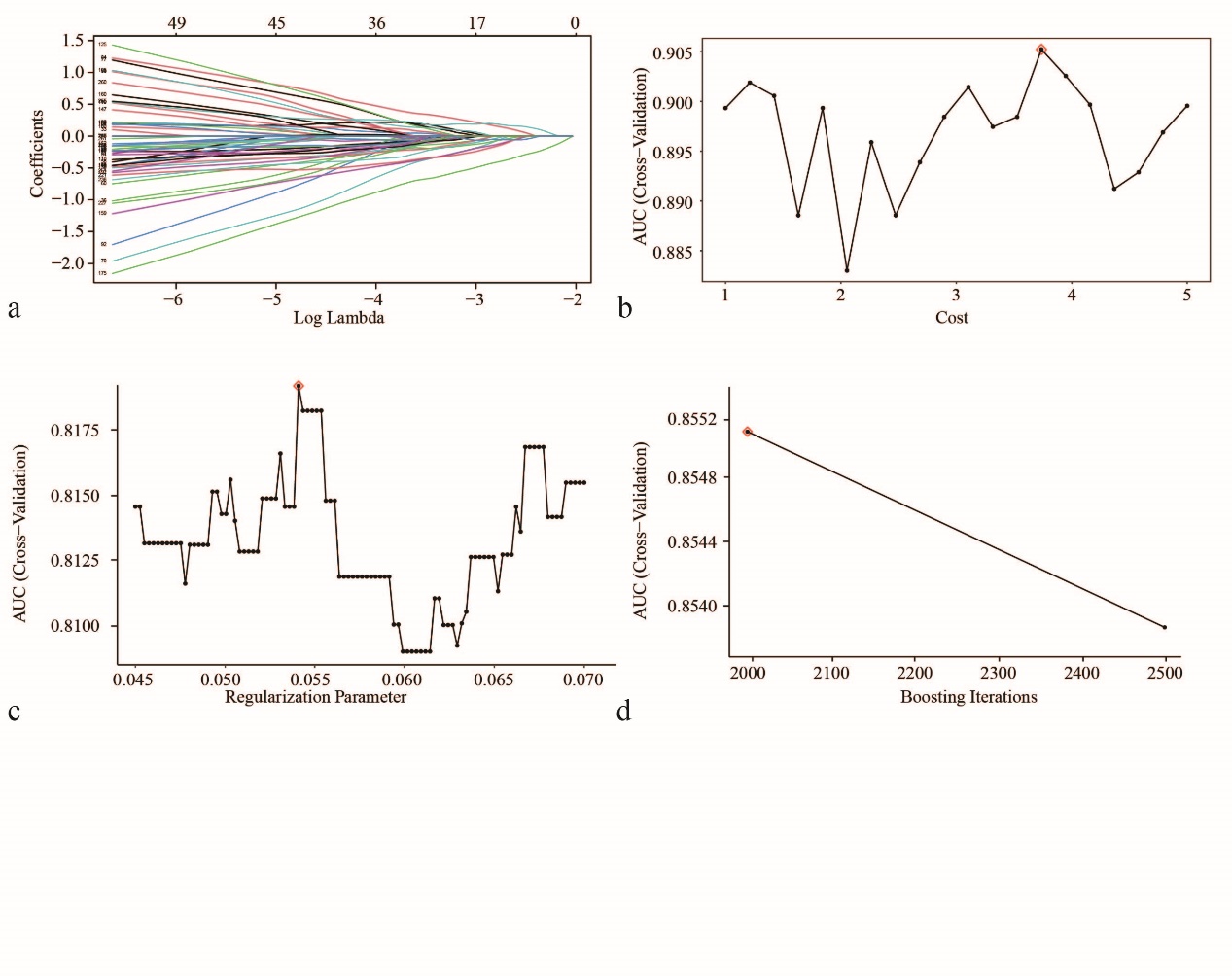


**Figure**.**S3 Model construction.** Process of model training in LASSO logistic regression model (a, c), supporting vector machine (SVM) model (c), and gradient boosting machine (GBM) model (d). (a) LASSO coefficient profiles of the selected radiomic features with non-zero coefficients at the optimal λ; (b) Selection of the best SVM model via cross-validation according to the criteria of AUC value; (c) Selection of the tuning parameter (λ) for the LASSO model via ten-fold cross-validation according to the criteria of AUC value; (d) Selection of the best GBM model via cross-validation according to the criteria of AUC value. For b, c, d, the models with the highest AUCs were outlined.


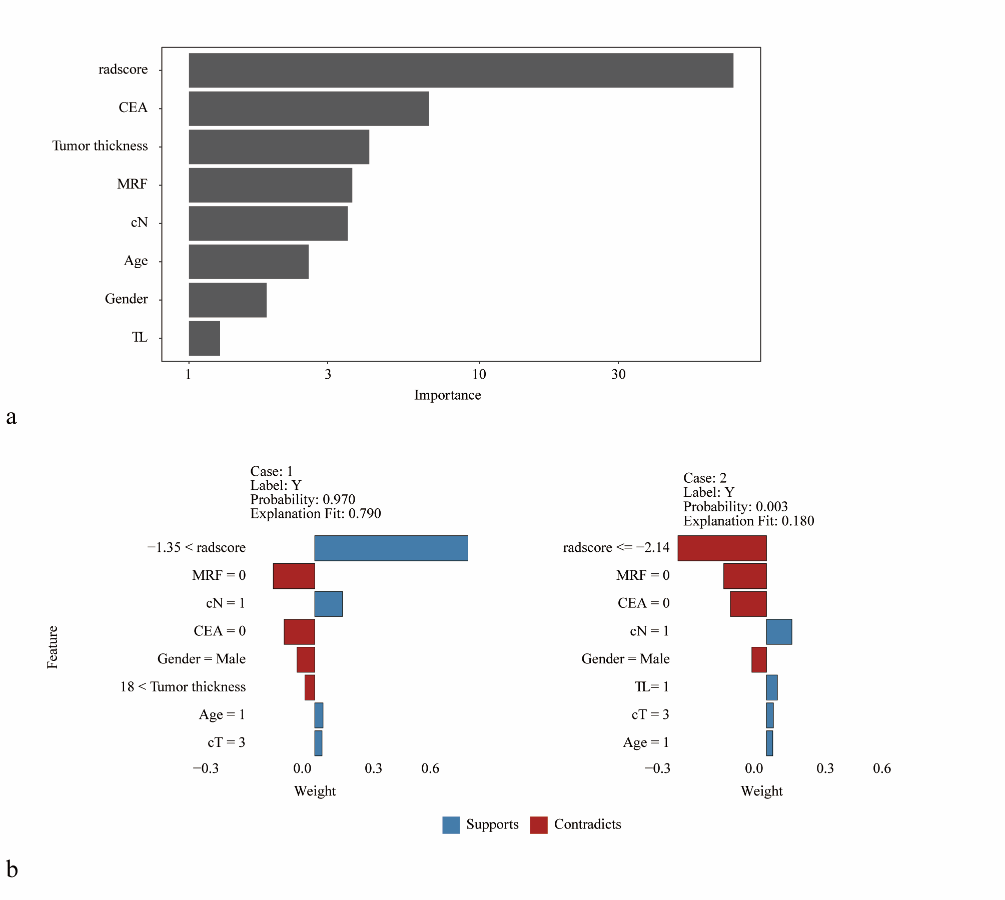


**Figure**.**S4 Detail information of the GBM models.** (a) the rank of importance of variables in the GBM models. (b) two cases of model prediction. Case 1 was a non-pCR patients and Case 2 was a pCR patients.

**Table.S4. Results of multivariate logistic regression analysis**

| Characteristic | *β* | OR (95% CI) | *P* |
| --- | --- | --- | --- |
| Intercept | 0.5721 |  |  |
| CT-base rad-score | 0.3375 | 1.401 (1.110-1.850) | 0.010 |
| MRI-based rad-score | 1.004 | 2.730 (1.327-6.419) | 0.011 |

*β*, regression coefficient; OR, odds ratio; CI, confidence interval.


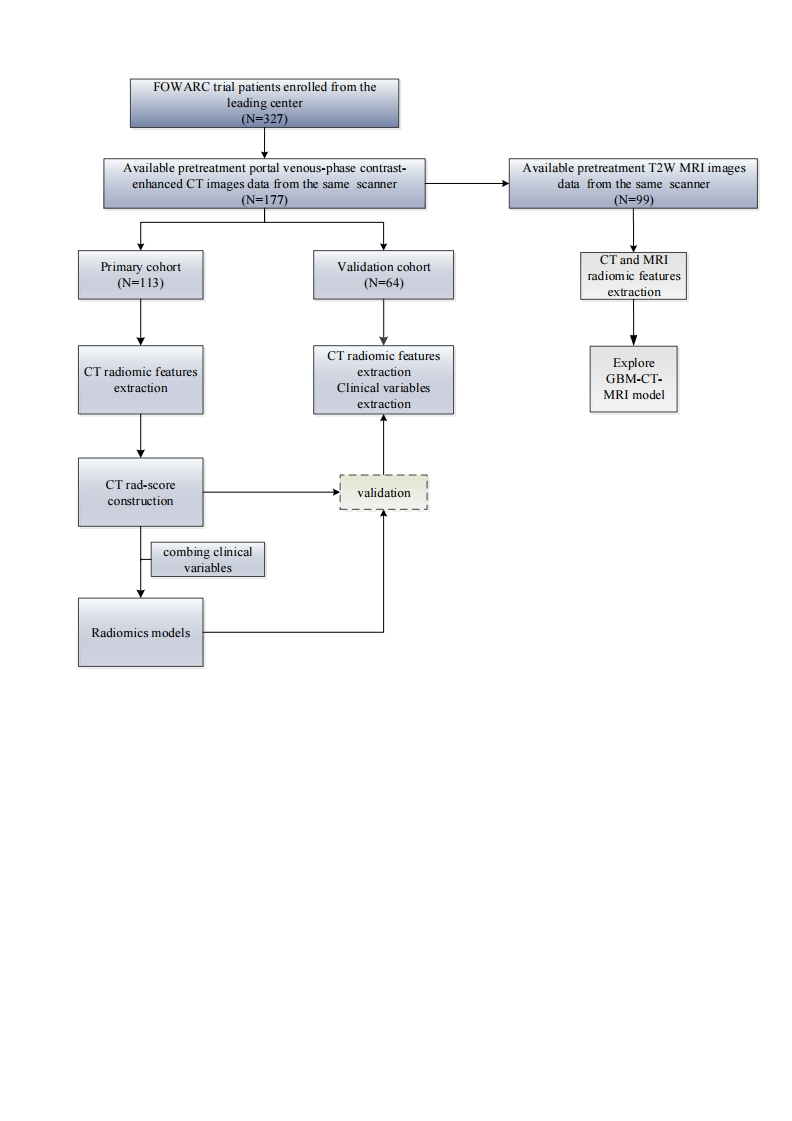


**Figure**.**S5 Patient disposition and the analysis protocol of the study.**
